# *Staphylococcus arlettae* mediated defense mechanisms and metabolite modulation against arsenic stress in *Helianthus annuus*

**DOI:** 10.3389/fpls.2024.1391348

**Published:** 2024-06-17

**Authors:** Muhammad Qadir, Anwar Hussain, Mohib Shah, Muhammad Hamayun, Amjad Iqbal, Muhammad Irshad, Ayaz Ahmad, Abdulwahed Fahad Alrefaei, Sajid Ali

**Affiliations:** ^1^ Department of Botany, Abdul Wali Khan University, Mardan, Pakistan; ^2^ Department of Food Science and Technology, Abdul Wali Khan University, Mardan, Pakistan; ^3^ Department of Biotechnology, Abdul Wali Khan University, Mardan, Pakistan; ^4^ Department of Zoology, College of Science, King Saud University, Riyadh, Saudi Arabia; ^5^ Department of Horticulture and Life Science, Yeungnam University, Gyeongsan, Republic of Korea

**Keywords:** arsenic, arsenic bioremediation, boosted antioxidant system, plant growth promotion, *Staphylococcus arlettae*

## Abstract

**Introduction:**

Arsenate, a metalloid, acting as an analog to phosphate, has a tendency to accumulate more readily in plant species, leading to adverse effects.

**Methods:**

In the current study, sunflower seedlings were exposed to 25, 50 and 100 ppm of the arsenic.

**Results:**

Likewise, a notable reduction (p<0.05) was observed in the relative growth rate (RGR) by 4-folds and net assimilation rate (NAR) by 75% of *Helianthus annuus* when subjected to arsenic (As) stress. Nevertheless, the presence of *Staphylococcus arlettae*, a plant growth-promoting rhizobacterium with As tolerance, yielded an escalation in the growth of *H. annuus* within As-contaminated media. *S. arlettae* facilitated the conversion of As into a form accessible to plants, thereby, increasing its uptake and subsequent accumulation in plant tissues. *S. arlettae* encouraged the enzymatic antioxidant systems (Superoxide dismutase (SOD), peroxidase (POD), ascorbate peroxidase (APX) and catalase (CAT)) and non-enzymatic antioxidants (flavonoids, phenolics, and glutathione) in *H. annuus* seedlings following substantial As accumulation. The strain also induced the host plant to produce osmolytes like proline and sugars, mitigating water loss and maintaining cellular osmotic balance under As-induced stress. *S. arlettae* rectified imbalances in lignin content, reduced high malonaldehyde (MDA) levels, and minimized electrolyte leakage, thus counteracting the toxic impacts of the metal.

**Conclusion:**

The strain exhibited the capability to concurrently encourage plant growth and remediate Ascontaminated growth media through 2-folds rate of biotransformation and bio-mobilization.

## 1 Introduction

As is regarded as the most harmful cancer-causing element and is graded as the fifth most potentially hazardous element (PHE) conferring to the Comprehensive Environmental Response, Compensation, and Liability Act (CERCLA) (Wang et al., 2020). As naturally ranks as the twentieth most abundant metal in the lithosphere (Patel et al., 2019) and acknowledged on a global scale for its harmful impacts on human well-being and environmental safety (Alka et al., 2020). Given that As contamination presents a significant risk to humans, animals, and plants, its remediation is of utmost importance (Masindi and Muedi, 2018). In certain parts of Asia, such as Bangladesh, China, India, and Pakistan, elevated levels of As in potable water have resulted in the emergence of areas known as ‘cancer villages’ (Cheng et al., 2019).

Governmental organizations like World Health Organization (WHO), the Agency for Toxic Substances and Disease Registry (ATSDR), Environmental Protection Agency (EPA) and International Agency for Research on Cancer (IARC) have indicated that extended exposure to As at a concentration of 10 μg/L could potentially lead to cancer (Atsdr, 2007; Marinho et al., 2019). The toxicity of As compounds can exhibit significant variation. Generally, As compounds can be categorized in terms of toxicity from highest to lowest as follows: inorganic As^+3^ > organic As^+3^ > inorganic As^+5^ > organic As^+5^ > elemental As (Gorby, 1988). As is believed to be introduced in substantial quantities into agricultural land and drinking water through both natural processes and human activities, impacting millions of individuals globally (Abbas et al., 2018). In certain countries across Asia and Europe, the pollution of As is notably widespread (Hering et al., 2017). Undoubtedly, this situation is deeply concerning, as the occurrence of As in drinking water, crops, and animal-derived products can lead to significant health problems in humans (da Silva et al., 2018; Suriyagoda et al., 2018; Yañez et al., 2019).

A range of amelioration technologies, encompassing both physicochemical and conventional methods, have been utilized to eliminate As from groundwater. However, conventional techniques, such as the application of fertilizers, only partially mitigate the adverse impacts of As (Mishra and Mishra, 2018), and this comes with the drawback of elevated operational costs (Singh et al., 2018). On the contrary, employing biological approaches can potentially yield significant As reduction at a lower expense. The utilization of plant species and microorganisms as bioremediators, a relatively recent but effective strategy, has gained prominence over the past few decades (Qadir et al., 2021). Various plants and microbial species possess the capability to bio-transform heavy metals from potentially toxic forms into non-toxic ones (Qadir et al., 2020; Husna et al., 2021). Microbial remediation is regarded as more efficient than phytoremediation in mitigating As and other heavy metal pollution. This approach is cost-effective, highly efficient, and generates fewer waste products (Qadir et al., 2022). Indeed, present-day investigations have demonstrated the impressive effectiveness of employing plant growth-promoting bacteria (PGPB) in areas contaminated with metals (Husna et al., 2022; Qadir et al., 2022).

The Environmental Protection Agency (EPA) classifies As as a potentially toxic contaminant within the division of heavy metals (Agency, 1989). The contamination of agricultural land with As poses a significant threat, as As compounds are highly mobile and can readily penetrate the food web (Hou et al., 2020). Given their notable mobility and carcinogenic properties, addressing the remediation of As-contaminated soil is imperative to safeguard agricultural land, boost crop yields, and fulfill the food requirements of the growing population (Bissen and Frimmel, 2003).

The unrestricted application of chemical fertilizers and the utilization of As-contaminated water for irrigation have introduced substantial quantities of As into agricultural soil. The excessive accumulation of As has rendered agricultural lands unproductive globally, necessitating urgent interventions. Sunflower (*Helianthus annuus*), an Asteraceae family member, holds economic significance due to its oil production and stands out as a accumulator of a number of heavy metals (Cutright et al., 2010). However, this property has led to its extensive use as an effective phytoremediator; yet, the process of hyperaccumulation notably diminishes its biomass (Roosens et al., 2003).

The primary aim of this research was to explore whether a specific bacterium found in the soil surrounding plant roots could enhance the removal of As while simultaneously promoting the growth of sunflowers. To achieve this goal, we set out to: (i) evaluate the arsenic tolerance of bacteria isolated from contaminated areas around plant roots; (ii) introduce the selected rhizobacteria to sunflowers by incorporating them into a Hoagland solution supplemented with arsenic; (iii) assess the arsenic uptake by sunflower seedlings associated with the rhizobacteria compared to those not associated with them; (iv) analyze the biochemical and physiological responses of sunflower seedlings in association with the chosen microbes as they accumulate arsenic, aiming to protect the host from its detrimental effects.

## 2 Materials and methods

### 2.1 Microbial strain selection

A strain of the rhizobacterium *Staphylococcus arlettae*, recognized for its resistance to heavy metals and its capability to promote crop growth, was acquired from the Plant Microbe Interaction Lab, Department of Botany at Abdul Wali Khan University Mardan (Qadir et al., 2020).

### 2.2 Screening the rhizobacterium for As tolerance and biotransformation

Luria Bertani broth was enriched with sodium arsenate at concentrations of 100, 300, 500, 900, and 1200 ppm to cultivate *S. arlettae*. The flasks containing LB broth with bacterial inoculation were placed in an incubator at 28°C and 120 rpm for 72 hours. Bacterial proliferation was assessed by measuring the optical density (OD) of the cultures at a wavelength of 600 nm. Additionally, the biotransformation potential of *S. arlettae* was evaluated by quantifying the presence of As^+5^ and As^+3^ in the bacterial strain’s culture supernatant exhausting the Community Bureau of Reference (BCR) technique (Kazi et al., 2005).

### 2.3 Screening the rhizobacterium for As biosorption and biotransformation

To achieve this objective, *S. arlettae* was cultured in a liquid medium, specifically Luria Bertani (L.B) broth, within a controlled environment at 120 rpm and a temperature of 28 °C for a duration of 24 hours. After the growth phase, the bacterial cells were segregated from the culture medium by centrifugation at 5600 rcf for 10 minutes. The isolated cells were later flushed with sanitized distilled water to remove any residual impurities. To deactivate cellular activities and enzymatic reduction processes, the cells were autoclaved at 121°C and 15 lbs of pressure for 15 minutes, rendering them inactive. The resulting heat-killed bacterial cells, devoid of enzymatic reduction capabilities, were mixed with As in Luria Bertani broth and incubated. As, in the form of sodium arsenate salt (Na_3_AsO_4_), was added at concentrations of 100, 300, 500, 900 and 1200 mg L^-1^. This incubation was conducted overnight at 28°C, facilitating interaction between the heat-killed cells and the As in the medium (Prasad et al., 2013). To facilitate the biotransformation of the metal, the chosen concentrations were increased in the culture medium and allowed to grow for duration of 24 hours at 28 °C. The concentrations of As species (As^+5^ and As^+3^) within the medium were assessed using the Community Bureau of References (BCR) technique. For the purpose of biotransforming the metal, the active *S. arlettae* culture was enriched with varying amounts of arsenate, as outlined previously. Subsequently, the bacterial culture, supplemented with the specified As concentrations, was incubated for a 24-hour period at a temperature of 28°C. Following the guidelines of the Community Bureau of References (BCR) protocol, the concentrations of both arsenate and arsenite were determined within the broth culture (Kazi et al., 2005).

### 2.4 Siderophore production

To determine siderophore production, a Chrome Azurol S (CAS) reagent was created by combining 121 mg of CAS with 100 mL of distilled water. Additionally, a ferric chloride solution (20 mL of 1 mM) was prepared using 10 mM HCl, and this solution was blended with the CAS solution. Alongside this, a solution of 20 mL hexadecyl trimethyl ammonium bromide (HDTMA) was formed by dissolving 729 mg of HDTMA in 400 mL of distilled water. For the quantitative assessment of siderophores, the bacterial culture, cultivated as detailed earlier, was spun down using a centrifuge at 10,000 rcf for 10 minutes to isolate the cell mass. Approximately 0.5 mL of the CAS reagent was merged with 0.5 mL of the culture supernatant, and the resulting mixture was left to incubate at room temperature for 20 minutes. Following the incubation, the absorbency of the solution was assessed at a wavelength of 630 nm (Arora and Verma, 2017). Siderophores produced by strain were quantified using the formula;


$$\text{\% Siderophore production }\left(\text{psu}\right)=\frac{\text{Ar}-\text{As}}{\text{Ar}}\times 100$$

Whereas,

*A*_r_ = optical density of CAS solution mixed with un-inoculated broth).

*A*_s_ = optical density of mixture of CAS solution and cell-free culture supernatant.

### 2.5 *H. annuus* seedlings bioassay

*H. annuus* seeds underwent surface disinfection using a solution of 0.1% HgCl_2_. After disinfection, the seeds were meticulously subjected to three rounds of washing with sterilized distilled water (dH_2_O) to eliminate any remaining disinfectant or contaminants (Younesikelaki et al., 2016). The sterilized *H. annuus* seeds were germinated in dampened sand under aseptic conditions. Once the young plants reached the point of having two leaves, they were transplanted into plastic pots with a capacity of 500 mL. Half-diluted Hoagland’s medium, a commonly chosen nutrient solution, was added to the plastic pots to provide essential nutrients for plant growth. The seedlings were positioned on a perforated thermopole sheet suspended above the nutrient solution. A steady stream of air was directed into the solution via a VEVOR Linear Air Pump (25W, 40W, 105W, 110V). A suspension containing 10 mL of *S. arlettae* bacterial cells, diluted to a concentration equivalent to 10^6^ cells mL^-1^, was applied to the *H. annuus* seedlings in 4 leaves stage. The experimental setup was planned according to a completely randomized block design, with a set of three replicates for each treatment. Within each replication, four *H. annuus* plants were included. The experiment incorporated two factors: *S. arlettae* inoculation and varying levels of arsenic (As^+5^) concentration, specifically 0, 25, 50, and 100 ppm. The plastic pots containing the *H. annuus* seedlings were maintained under sterile and controlled conditions within a LabTech Model: LGC-5101 G growth chamber. These conditions encompassed a temperature of 28.2°C, a photoperiod of 13 hours, and a humidity level of 68%. After a cultivation period of 20 days, when the *H. annuus* seedlings had progressed to the 4–5 leaf stage, they were harvested for subsequent analysis and assessment. The duration of the experiment and the selection of growth stages were chosen to capture the response of the seedlings to the combined influences of *S. arlettae* inoculation and varying levels of As-induced stress.

### 2.6 Seedlings growth parameters

Various growth parameters were measured from the collected plants. The relative growth rate (RGR) (Hoffmann and Poorter, 2002), net assimilation rate (NAR) (Vernon and Allison, 1963), *S. arlettae* colonization and other growth characteristics were documented upon conclusion of the experiment.


$$RGR\;=\frac{\ln W2\;\;–\ln W1\;}{t\;2\;\;–\;t\;1\;}$$

In which, the natural logarithm (ln) is utilized, while W1 and W2 represent the dry weights of the plants at times t1 and t2.


$$NAR=\frac{W\;2\;-W\;1}{t\;2\;-t\;1\;}\times \frac{In\;L\;2\;-In\;L\;1\;}{L\;2\;-L\;1\;}$$

In the experimental context, W2 signifies the dry weight of the plants at time t2, and W1 represents the dry weight at time t1. Likewise, ln L2 and ln L1 correspond to the natural logarithms of leaf areas L2 and L1 at times t2 and t1, respectively.

The examination was repeated thrice, and the collected data were combined and subsequently subjected to thorough statistical analysis.

### 2.7 Estimation of indole acetic acid in plant biomass

The fresh leaves (mature leaves) of the host plant, which were utilized for the analysis of plant hormones, underwent a preparation process that involved grinding using liquid nitrogen. To extract indole acetic acid, 1 gram of powdered mature plant leaves (or 1 mL of plant exudates) was homogenized in 100% methanol at a ratio of 2 milliliters per gram of plant sample. Following this, centrifugation was performed at a 5600 rcf. The extracted IAA was collected from the centrifuged material, and the resulting supernatant was utilized to quantify its concentration using the Salkowski reagent technique (Salkowski reagent consisted of a mixture of 15 mL 0.5 M FeCL_3_, 500 mL distilled water, and 300 mL of concentrated H_2_SO_4_. Approximately, 1 mL of samples were mixed with 2 mL of Salkowski reagent and kept for incubation for at least 30 minutes and optical density was recorded at 540 nm). This established method is exploited to determine the quantity of IAA present in plant samples (Hussain and Hasnain, 2011).

### 2.8 Estimation of sugar and lipids

By either blending 0.1 g of fresh plant leaves or mixing 0.1 mL of leaf exudates with 1 mL of a solution composed of methanol, chloroform, and water in equal parts (1:1:1 ratio), soluble sugars were extracted. The samples were thoroughly mixed using a vortex mixer and then allowed to incubate for 30 minutes in a water bath set at 50°C. Following incubation, the samples were subjected to centrifugation at 14,000 rcf for 5 minutes at room temperature. The assessment of complete soluble sugar concentration was carried out exhausting the phenol-sulfuric acid technique (Mohammadkhani and Heidari, 2008). In short, 100 μL of supernatant was mixed with 1 mL of 80% phenol and then incubated at room temperature for 10 minutes. The mixture was then treated with 5 mL of saturated H_2_SO_4_, vortexed and incubated for 60 minutes at ambient conditions. The optical density was finally measured at 485 nm, the sugar levels were depicted as μg.g^-1^.

To assess the lipid contents, 0.2 g of fresh leaves were homogenized immediately in 2 mL of 1:2 methanol: chloroform (v/v) solution. The blend was vortexed for a few seconds and then filtered. To the filtrate, 0.8 mL of the 0.73% NaCl was added. The lower phase containing lipids were collected. The phase with lipids was isolated into a distinct tube and the vanillin-phosphoric acid reagent technique was used for its quantification. In brief, the fraction with lipids was then dried for 5 hours using an air flux condenser over a water bath set at 50–60°C. Highly concentrated sulfuric acid (5 mL) was integrated with 0.2 mL of the samples, followed by mixing for 10 minutes. After the addition of phosphoric-vanillin reagent (2.4 mL), the absorbance was registered at 490 nm (Van Handel, 1985).

### 2.9 Reactive oxygen species visualization using diaminobenzidine assay

With slight modifications, we employed the 3,3′-diaminobenzidine (DAB) protocol in accordance with the method outlined by O’Brien et al. (2012). Leaf sections from the plants were washed and circular pieces were immersed in 2 mL of DAB stain. Subsequent to this, the samples were transferred to a shaking incubator for incubation at 80–100 rpm for a duration of 4–5 hours. Following the incubation under the mentioned conditions, the leaf specimens were subjected to a bleaching solution (ethanol: acetic acid: glycerol; 3:1:1) for an hour in a water bath. This bleaching step was carried out to remove photosynthetic pigments, thereby revealing any DAB staining that might be present. Subsequently, we assessed the concentration of ROS in the leaf samples.

### 2.10 Estimation of non-enzymatic antioxidant potential

To extract flavonoids, 500 mg of fresh leaves from the host plant were ground and mixed with 5 mL of 80% ethanol. Alternatively, 0.5 mL of root exudates was used. The mixture was allowed to rest overnight in a shaker. Afterward, centrifugation was conducted at 5600 rcf at a temperature of 25°C for a duration of 15 minutes. The resulting extract was then shifted to a new 50 mL Falcon tube and kept at 4°C. The estimation of total flavonoid content was conducted using the AlCl_3_ technique, as previously mentioned (El Far and Taie, 2009).

To extract total phenolic contents, 1 g of seedlings (or 1 mL of exudates) were integrated in 16 mL of ethanol and incubated at incrementally increasing temperatures (from 20°C to 80°C) for a duration of 3 hours. Following incubation, the mixture underwent centrifugation at 5600 rcf for 10 minutes at room temperature. The resulting supernatants were filtered and their quantity was decreased to 1 mL using a rotary evaporator, maintaining a temperature of 40°C. The concentrated extracts were diluted using 10 mL of distilled water (dH_2_O) and stored at 4°C until further use. Phenolic content was assessed using the previously outlined method (Malik and Singh, 1980).

The extraction of proline content involved crushing 0.2 g of leaves into 1 mL of a water/ethanol solution (60:40 v/v), or using 0.2 mL of root exudates. The resulting homogenate was left overnight at 4°C and then subjected to centrifugation at 5600 rcf for 5 minutes. This extraction process was conducted once for 100% extraction, although the initial extraction already yielded 93%. The examination of proline content followed a well-established procedure (Bates et al., 1973).

To extract glutathione (GSH), fresh leaves from the host seedlings were employed. The extraction procedure began with homogenizing the leaves in 100% methanol, ensuring effective mixing and disruption of the plant tissue. Following this, the homogenate underwent sonication for a period of 15 minutes (Muryanto et al., 2017). The measurement of GSH activity was carried out using a reaction mixture comprising of 1.8 mL phosphate buffer, along with 300 μL each of EDTA, NADPH, oxidized glutathione (GSSG), and enzyme extract (1 mg mL^-1^). The calculation involved assessing the oxidation of NADPH at 340 nm. The decrease in absorbance per minute was recorded at a wavelength of 340 nm (Carlberg and Mannervik, 1975).

### 2.11 Estimation of enzymatic antioxidant potential

The assessment of free radical scavenging activity in plant material was conducted using DPPH (1, 1-diphenyl-2-picrylhydrazyl) as the free radicals, sticking to the method by Ahmad et al. (2011)_ENREF_2. Fresh plant leaves (100 mg) were pulverized with 1 mL of methanol, while a DPPH standard solution (0.004%) was prepared by dissolving 0.004 g of DPPH in the chosen solvent (methanol). For the determination of activity, 1 mL of the plant sample was merged with 2 mL of freshly prepared DPPH solution, and the mixture was kept in the absence of light for 30 minutes. The absorbance was subsequently quantified at 517 nm. A reduction in color intensity and absorption indicated radical scavenging, which was assessed employing the subsequent equation:


$$\%DPPH=\left(\frac{1-AE}{AD}\right)\times 100$$

In the above equation;

The AE represents absorbance of DPPH blended with plant sample,

The AD represents absorbance of DPPH solution in methanol only.

The primary rate of H_2_O_2_ hydrolysis was utilized to determine CAT activity, pursuing the procedure laid by Radhakrishnan and Lee (2013). For the extraction, 1 g of fresh leaves was mashed in 10 mL of phosphate buffer (pH 7). The resulting homogenates were then subjected to centrifugation at 10000 rcf for 20 minutes at 30 °C. In a mixture of 2.6 mL of 0.05 M PBS (phosphate buffer, pH 7), 0.4 mL of 3% H_2_O_2_, 100 µM EDTA, and 100 µL of the supernatant from the leaf extract were mixed. The reduction in absorbance at 240 nm was computed as the hydrolysis of μM H_2_O_2_ per minute.

Following the methodology established by Asada (1992), APX activity was determined. To prepare the extract, 1 g of fresh leaves was crushed in 10 mL of PBS (pH 7). The homogenates were then subjected to centrifugation at 10000 rcf for 20 minutes at 30°C. In a sample volume of 0.2 mL of the supernatant, 100 µL of hydrogen peroxide (H_2_O_2_) at a concentration of 0.1 mM was introduced. Subsequently, 0.6 mL of phosphate-buffered saline (PBS) with a concentration of 50 mM at a pH of 7.0, along with 0.1 mL of ascorbic acid at a concentration of 0.5 mM, was added. The reduction in absorbance was monitored at 290 nm and reported as U mg^-1^ protein, where U signifies the change in 0.1 absorbance units per minute per milligram of protein.

POD enzymatic activity of leaf extracts was determined through the dehydrogenation of a substrate, guaiacol, following the procedure by Malik and Singh (1980). The enzyme was procured from host plant leaves in 3 mL of 0.1 M phosphate buffer (pH 7.0). To start with, 100 mg of fresh leaves were pulverized with 1 mL of phosphate buffer using a mortar and pestle. Consequent to this, the resulting mixture was processed through centrifugation at 5°C and 12,000 rcf for 15 minutes. For the assay, 100 µL of supernatant was introduced into the cuvette, and then 3 mL of 0.1 M PBS, 30 µL of H_2_O_2_ (12.3 mM or 0.04%), and 50 µL of guaiacol (20 mM). The subsequent increase in optical density at 436 nm was recorded, and the calculation was performed as follows:


$$\text{Enzyme activity}=\left(\frac{500}{\text{Δt}}\right)\times \left(\frac{1}{1000}\right)\times \left(\frac{\text{TV}}{\text{VU}}\right)\times \left(\frac{1}{\text{f wt}}\right)$$

Whereas;

In the given equation, Δt represents the variation in time, TV denotes the total amount of the prepared sample, VU represents the volume used for analysis, and f wt. signifies the fresh weight of the sample in grams.

The leaf superoxide dismutase (SOD) activity was assessed by its capability to hinder the photochemical reduction of nitro blue tetrazolium (NBT), as outlined by Dhindsa et al. (1981). The sample volume for optical density measurement consisted of 2.725 mL, which included 50 mM PBS (pH 7.8), 750 μM NBT, 20 μM riboflavin, 26 mM methionine, and 1 μM EDTA. Approximately 25 μL of plant extract and 250 μL of distilled water (dH_2_O) were mixed and exposed to white light with an intensity of 4,000 Lux for 15 minutes. Following this, the absorbance was recorded at 560 nm.

### 2.12 Determination of malondialdehyde contents

The methodology explained by Schmedes and Hølmer (1989) was utilized to estimate malonaldehyde content. In this process, 200 mg of leaves were homogenized in 2 mL of 0.6% thiobarbituric acid. Centrifugation of the resulting mixture was performed for 10 minutes at 8064 rcf, and then heated at 100°C in a water bath for 15 minutes. After reaching ambient temperature, a repeat centrifugation at 8064 rcf was performed on the mixture. The absorbance was quantified at wavelengths of 532 nm, 600 nm, and 450 nm. A standard graph was created using established concentrations of malonaldehyde (MDA).

### 2.13 Electrolyte leakage

To eliminate surface-attached electrolytes, leaves of 7-day old *H. annuus* plants were rigorously flushed with deionized water (dH_2_O). Subsequently, the leaves were placed in a vial with a cover, containing 10 mL of deionized water. This vial was then incubated at a temperature of 25°C in a shaking incubator operating at 120 rpm. After 24 hours of incubation, the initial electrical conductivity (L1) was calculated. Afterward, the samples were subjected to autoclaving at 120°C for 20 minutes, and the final electrical conductivity (L2) was deliberated at 25°C (Lutts et al., 1996). The electrolyte leakage was determined using the following equation:


$$EC=\frac{L1}{L2}\times 100$$

### 2.14 Estimation of root lignin contents

The estimation of root lignin content was conducted by means of amended Kolson guideline (Moreira-Vilar et al., 2014). Root fragments (1 g) were subjected to a treatment at 47°C with 72% H_2_SO_4_ and agitation for a duration of 7 minutes. After digestion, the treated root fragments were sterilized by autoclaving at 121°C for 30 minutes. Subsequent to autoclaving, the mixture was filtered to partition the soluble and insoluble lignin fractions. The absorbance of the soluble fraction was quantified at wavelengths of 280 nm and 215 nm. The content was subsequently computed using the provided formula:


$$\text{S}=\frac{4.53\left(A215-A280\right)}{300}$$

The above-mentioned equation was derived from these two equations: A215 = 0.15 F+70 S and A280 = 0.68 F+18 S.

Whereas,

A_280_= Absorption at 280 nm, A_215_= Absorption at 215 nm, F=The furfural (g), S=The soluble lignin (g).

The absorbance values for furfural and soluble lignin at 280 nm and 215 nm were measured as 0.68, 0.15 and 18, 70, respectively. The insoluble residues were subjected to combustion at 550°C for a duration of 4 hours to determine the ash content. The complete lignin content was ascertained by summing the insoluble and soluble lignin fractions (mg g^-1^ of cell wall), and then incorporating this sum with the non-soluble residues, derived from the disparity between the remaining mass and ash content.

### 2.15 Estimation of As via sequential extraction

As level was calculated in samples by means of the established BCR protocol (Kazi et al., 2005). The process was carried out in three phases:

Phase 1: Exchangeable and Acid Soluble.

Using 0.5 g of air-dried samples, 20 mL of 0.11M acetic acid (CH_3_COOH) was added and stirred for a period of 12–24 hours at temperatures ranging from 25 to 30 °C. To separate the residues, the resulting extract was subjected to centrifugation at 3000 rcf for a duration of 20 minutes at 30 °C.

Phase 2: Reducible.

The residue obtained from phase 1 was combined with 20 mL of 0.5 M NH_2_OH–HCl (hydroxylamine-hydrochloride) and subjected to agitation for a duration of 16 hours at temperatures between 25 and 30°C. The mixture’s pH was lowered to 1.5 using nitric acid (HNO_3_).

Phase 3: Oxidizable.

The mixtures from Step 2 were combined with 30% H2O2 (10 mL), and these resultant mixtures were incubated for an hour in a shaking incubator. The pH was adjusted to 2 using NaOH. The contents were then dried and mixed with 1M ammonium acetate (25 mL) and subjected to further shaking for a period of 16 hours.

Following the extraction process, the residue was rinsed with ultra-pure water and placed in a shaking incubator for 15 minutes. Subsequently, the mixture was centrifuged for a duration of 20 minutes at a speed of 3000 rcf.

### 2.16 Metal determination in treated samples

Atomic absorption spectroscopy (Perkin Elmer Analyst 700, USA) was employed to examine the samples. Air/acetylene flame was used according to producer’s recommendations.

### 2.17 Bioconcentration factor

The metal in Hoagland’s medium and plants biomass were ascertained through the following formula:


$$BCF=\frac{Metal\;concentration\;in\;biomass}{Metal\;concentration\;added\;to\;Hoagland's\;media}$$

### 2.18 Analysis of the data

The experiments were conducted in triplicate, and the collected data was subjected to analysis of variance (ANOVA) using IBM SPSS Statistics 21. Significant differences among means were determined using Duncan’s Multiple Range Test (DMRT) at a significance level of p<0.05. Graphs were generated using GraphPad Prism software (Version 5.03).

## 3 Results

### 3.1 Effects of As on bacterial mass, biosorption, biotransformation and siderophore production

**Figure 1:**
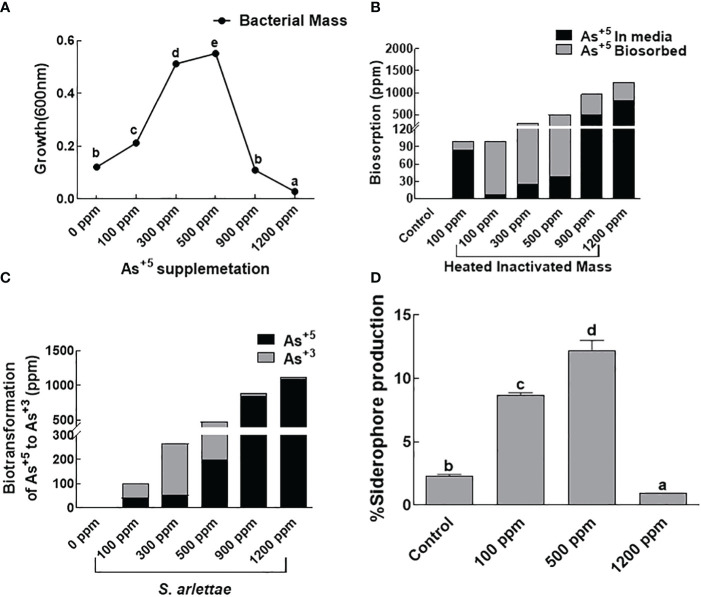
Effect of arsenate supplementation on **(A)** *S. arlettae* growth, **(B)** biosorption, **(C)** biotransformation of As^+5^ to As^+3^ and **(D)** siderophores production. The bars in the figure represent the mean values of the data, with error bars indicating the standard error (± SE). The letters displayed on the bars indicate the significance levels, with significance denoted at p<0.05.

The bacterial isolate *S. arlettae*, treated with sodium arsenate (Na_3_AsO_4_) as the source of As, demonstrated an increase in biomass with the rise in As^+5^ concentration up to 500 ppm (**Figure 1A**). However, a decline in bacterial biomass production was observed beyond 500 ppm. The biosorption capacity of the bacterial strain also increased with the elevated levels of As^+5^. Active biosorption was observed up to 500 ppm of As^+5^. No significant increase or decrease in biosorption potential was noted in the bacterial host at 900 and 1200 ppm of As^+5^ (**Figure 1B**). A comparable pattern was noted for biotransformation, with maximum biotransformation of As recorded in media supplemented with 500 ppm of As^+5^. The biotransformation capacity of *S. arlettae* declined in media holding 900 and 1200 ppm of As^+5^ (**Figure 1C**). Regarding siderophores, the rhizobacteria *S. arlettae* exhibited augmented activity when encountering 500 ppm As^+5^ stress (**Figure 1D**).

### 3.2 Modulating host physiology and biochemistry

#### 3.2.1 Growth parameters

**Figure 2:**
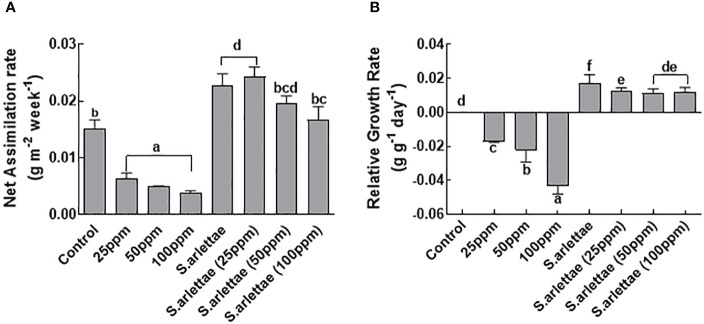
Role of As^+5^ and *S. arlettae* on **(A)** net assimilation rate (NAR) and **(B)** relative growth rate (RGR) of host. The bars in the figure represent the mean values of the data, with error bars indicating the standard error (± SE). The letters displayed on the bars indicate the significance levels, with significance denoted at p<0.05.

Exposure of *H. annuus* to 100 ppm of As stress resulted in a significant 75% reduction in net assimilation rate (NAR) compared to untreated plants (**Figure 2A**). The presence of the rhizobacterial strain *S. arlettae* positively influenced NAR. Notably, plants inoculated with *S. arlettae* and treated with As exhibited a higher NAR compared to untreated plants (**Figure 2A**).

As stress also had a significant impact (P=0.05) on the relative growth rate (RGR) of *H. annuus*, mirroring the NAR trend (**Figure 2B**). Plants exposed to 100 ppm of As stress experienced a roughly 4-fold reduction in RGR compared to control plants. Following inoculation with *S. arlettae*, the RGR of host plants increased by approximately 8-fold under 100 ppm As stress. The maximum RGRs observed in *S. arlettae*-inoculated plants exposed to 25 ppm As stress, while non-inoculated *H. annuus* plants supplemented with 100 ppm As exhibited lower RGR (**Figure 2B**).

### 3.3 Host metabolites

#### 3.3.1 Indole acetic acid

**Figure 3:**
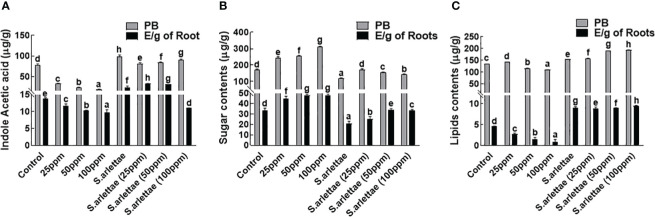
Role of As^+5^ and *S. arlettae* on **(A)** indole acetic acid, **(B)** soluble sugars and **(C)** lipids of host. In the figure, “PB” represents fresh plant biomass, and “E/g of Roots” indicates exudates per gram of roots. The bars in the figure represent the mean values of the data, with error bars indicating the standard error (± SE). The letters displayed on the bars indicate the significance levels, with significance denoted at p<0.05.

*H. annuus* seedlings were found to synthesize 85.7% of indole acetic acid (IAA), predominantly through endogenous processes (**Figure 3A**). The introduction of As stress had a notable impact (p<0.05) on IAA production and its endogenous deposition, resulting in a reduction to 20.88% compared to untreated plants. Furthermore, a decline in IAA production (from 14.12% to 10.61%) was observed in *H. annuus* seedlings experiencing heightened As levels. The inoculation of *S. arlettae* improved the seedlings’ capability for IAA synthesis and led to significant (P=0.05) endogenous accumulation. However, a more pronounced reduction (12.42%) in exogenous IAA content was noted within the host plants supplemented with 100 ppm of As^+5^.

#### 3.3.2 Sugars and lipids

Elevated metal concentrations in the growth medium resulted in an enhancement of both endogenous and root-exuded sugar contents in *H. annuus* seedlings (**Figure 3B**). After being exposed to 100 ppm of As^+5^, the intrinsic sugar content of *H. annuus* seedlings increased by 68.23%, while in root exudates, there was a 7.08% increase compared to control seedlings. However, the inoculation of *H. annuus* plants with *S. arlettae* led to a reduction in seedling sugar contents as As^+5^ stress increased. In the *S. arlettae*-inoculated *H. annuus* seedlings, there was a decrease of 13.86% in endogenously accumulated sugars and 0.11% in root-exuded sugars following treatment with 100 ppm of As^+5^.

The accumulated lipid contents of the host seedlings showed notable changes in response to As-induced stress. With increased levels of As^+5^ in the half-strength Hoagland’s media, the concentration of endogenous lipids continued to decrease (**Figure 3C**). However, when plants were inoculated with the selected PGPR, a notable rise was observed in the overall lipid concentration. This indicated a substantial improvement of approximately 70.21% after exposing the plants to 100 ppm of As^+5^, compared to the control seedlings.

### 3.4 Rhizobacterial mediated antioxidant response

#### 3.4.1 Nonenzymatic antioxidants

**Figure 4:**
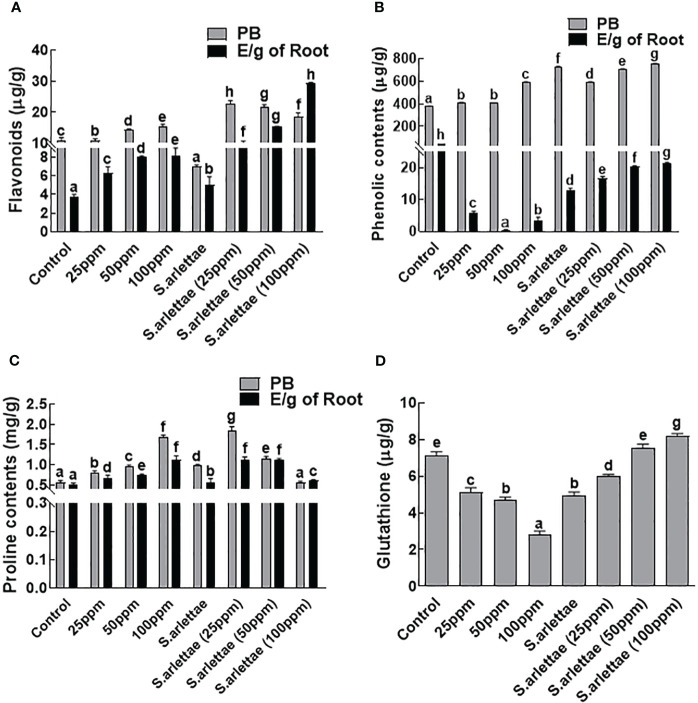
Role of As and *S. arlettae* on **(A)** total flavonoids, **(B)** total phenolics, **(C)** proline and **(D)** glutathione in host biomass (PB) and their exudates (E/g of root). The bars in the figure represent the mean values of the data, with error bars indicating the standard error (± SE). The letters displayed on the bars indicate the significance levels, with significance denoted at p<0.05.

The *H. annuus* seedlings exhibited substantial production of flavonoids, which accumulated endogenously in various plant parts. Furthermore, significant amounts of flavonoids were released by the *H. annuus* roots in their vicinity (**Figure 4A**). Notably, there was a significant (P=0.05) rise of 98.60% in endogenous flavonoid content and 55.05% in root-exuded flavonoids after subjecting the *H. annuus* seedlings to 100 ppm of As-induced stress. Inoculating *H. annuus* seedlings with *S. arlettae* further enhanced the flavonoid contents, resulting in a 51.79% increase in endogenous flavonoids and an impressive 174.10% increase in root exudates. The highest levels of endogenous (126.21%) and root-exuded (199.67%) flavonoids were observed in the *S. arlettae*-associated *H. annuus* plants experiencing 100 ppm of As stress.

The accumulation of endogenous phenolic contents in *H. annuus* seedlings exhibited a decrease of 33.49% under the influence of 100 ppm As^+5^ stress relative to the untreated plants (**Figure 4B**). In contrast, the exogenous phenolic contents of *H. annuus* seedlings increased by 5.94% as the As^+5^ stress level increased. Co-cultivation of the host plants with *S. arlettae* resulted in an enhancement of the total phenolic content in *H. annuus* seedlings by 25.61% relative to the non-inoculated seedlings. Conversely, a decrease of 3.13% was observed in the exogenous phenolics of the *H. annuus* plants treated with 100 ppm As^+5^ stress.

Significant amounts of free amino acids, such as proline, were produced by the *H. annuus* plants, with a substantial quantity (52.12%) stored endogenously and a portion (47.87%) released through the roots into the rhizosphere (**Figure 4C**). The induction of As stress significantly (P=0.05) enhanced the plants’ endogenous (102.63%) and root-exuded (56.76%) proline contents compared to the control seedlings. Additionally, the association with *S. arlettae* resulted in a slight decrease in endogenous proline accumulation by 0.5%, while higher amounts of proline (8.32%) were released through the roots upon exposure to 100 ppm of As stress (**Figure 4C**).

Similarly, in the case of glutathione, the *H. annuus* seedlings treated with the aforementioned levels of As^+5^ exhibited a decline. However, after inoculating the *H. annuus* seedlings with *S. arlettae*, the glutathione levels increased with the elevation of As^+5^ concentration in the media (**Figure 4D**).

#### 3.4.2 Antioxidant response

**Figure 5:**
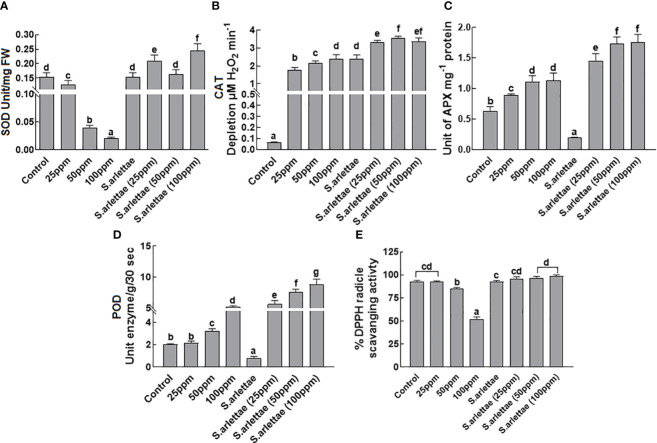
Effect of As and *S. arlettae* on **(A)** superoxide dismutase (SOD) **(B)** catalase (CAT) **(C)** ascorbate peroxidase (APX) **(D)** peroxidase (POD) and **(E)** %DPPH radicle scavenging activity of *H. annuus* seedlings. Data were collected from 20 days stressed plants. The bars in the figure represent the mean values of the data, with error bars indicating the standard error (± SE). The letters displayed on the bars indicate the significance levels, with significance denoted at p<0.05.

When *H. annuus* seedlings were exposed to the selected concentrations of the metal, there was a considerable decrease in SOD activity, with a sharp decline between 50 to 100 ppm of As^+5^ stress (**Figure 5A**). However, the application of *S. arlettae* to As*^+5^* stressed *H. annuus* seedlings led to an improvement in SOD contents at all tested As^+5^ concentrations. Remarkably, a significant (P=0.05) rise in SOD activity was detected in the host seedlings experiencing 100 ppm of As^+5^ stress (**Figure 5A**).

The catalase activity in *H. annuus* seedlings increased with higher levels of As^+5^ stress (**Figure 5B**). The association of *S. arlettae* with *H. annuus* seedlings further elevated the catalase levels in the host plants under As^+5^ stress. A similar trend was observed for ascorbate peroxidase (APX) activity when *H. annuus* seedlings were exposed to As^+5^ stress (**Figure 5C**). Interestingly, inoculation of *H. annuus* seedlings with *S. arlettae* reduced APX activity significantly under normal conditions (without As^+5^ stress). However, under As^+5^ stress, APX activity surged sharply (**Figure 5C**).

Exposure of *H. annuus* to As^+5^ stress led to an increase in peroxidase activity, corresponding to the elevated levels of As^+5^ in the media (**Figure 5D**). Co-cultivation of *H. annuus* seedlings with the rhizobacterial strain *S. arlettae* further intensified the peroxidase activity in the host seedlings under As^+5^ stress. Remarkably, *S. arlettae*-associated seedlings exhibited approximately a 3-fold increase in peroxidase activity compared to non-associated seedlings exposed to 100 ppm of As^+5^ stress (**Figure 5D**).

The activity of radical scavenging was computed following exposure of the plants to As^+5^ stress as described earlier. With elevated levels of As^+5^ in the medium, the radical scavenging activity decreased (**Figure 5E**). In contrast, *H. annuus* seedlings inoculated with the rhizobacteria *S. arlettae* and subjected to As^+5^ stress exhibited enhanced radical scavenging activity. Higher radical scavenging es were recorded in *S. arlettae*-inoculated *H. annuus* seedlings under 50 and 100 ppm of As^+5^ stress.

### 3.5 Malonaldehyde contents, electrolyte leakage and root lignification

**Figure 6:**
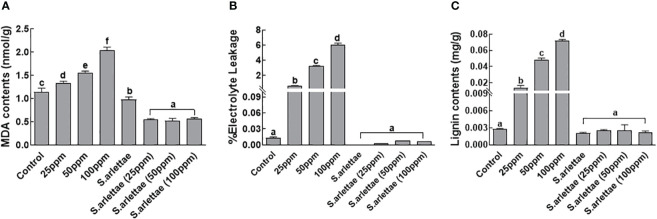
Role of As and *S. arlettae* on **(A)** MDA, **(B)** electrolyte leakage and **(C)** lignification in *H. annuus* seedlings. The bars in the figure represent the mean values of the data, with error bars indicating the standard error (± SE). The letters displayed on the bars indicate the significance levels, with significance denoted at p<0.05.

A rising trend was observed during the assessment of MDA (malondialdehyde) contents in *H. annuus* seedlings under increasing As^+5^ stress (**Figure 6A**). However, this trend was reversed when the plants were inoculated with *S. arlettae* and subjected to As^+5^ stress. Similar trends were noticed for electrolyte leakage when *H. annuus* seedlings were exposed to As^+5^ stress in a concentration-dependent manner (**Figure 6B**). Interestingly, in *S. arlettae*-treated host seedlings, the electrolyte leakage was comparable to that of the control seedlings, which were *H. annuus* seedlings without undergoing As^+5^ stress. The lignin contents nearly doubled after treating the host plants with elevated levels of As^+5^ (**Figure 6C**). Conversely, there was a sharp reduction in lignin observed in *S. arlettae*-associated *H. annuus* seedlings experiencing various doses of As^+5^ stress, and these levels were comparable to those of the control seedlings.

### 3.6 Accumulation/biotransformation of As and bioconcentration factor

**Figure 7:**
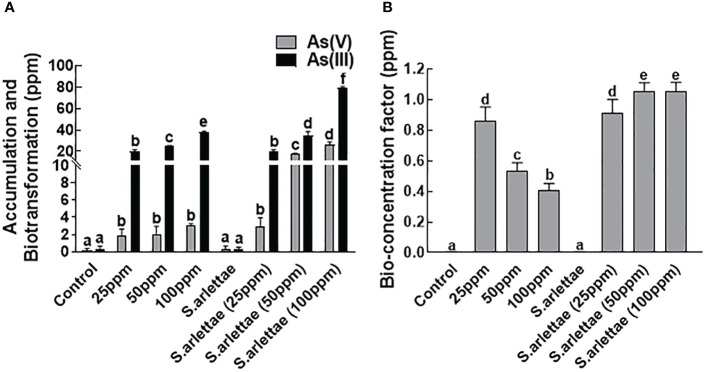
Effect of As and *S. arlettae* on **(A)** accumulation and biotransformation and **(B)** Bioconcentration factor. The bars in the figure represent the mean values of the data, with error bars indicating the standard error (± SE). The letters displayed on the bars indicate the significance levels, with significance denoted at p<0.05.

Cultivating *H. annuus* seedlings in Hoagland’s medium supplemented with the mentioned concentrations of As revealed a notable increase in both the accumulation and biotransformation of As, converting it from the As^+5^ to As^+3^ form (**Figures 7A, B**). As accumulation in *H. annuus* seedlings displayed a direct correlation with the rise in As^+5^ concentration in the medium, peaking at 100 ppm of arsenate supplementation.

In a similar vein, the inoculation of the host plants with *S. arlettae* resulted in the restoration of host plant growth across all metal concentrations, evident by higher NAR and RGR (**Figures 2A, B**). Furthermore, when *H. annuus* seedlings were co-cultivated with *S. arlettae*, the absorption, conversion, and accumulation of As were notably boosted, nearly doubling compared to the untreated control plants, as illustrated in **Figures 7A, B**.

## 4 Discussion

The shift of modern society toward industrialization has disrupted ecosystems, with one of the most concerning issues being the disposal of waste into irrigation water. This practice results in the buildup of pollutants, especially heavy metals, in agricultural lands. Among these metals, As stands out due to its high toxicity. In this study, we examined the response of a rhizobacterium to high levels of As exposure. Our results disclosed that the rhizobacterium *S. arlettae* displayed rapid growth when subjected to As concentrations of up to 500 ppm. This strain demonstrated superior growth in the presence of As, predominantly up to 500 ppm. The proficiency of *S. arlettae* to bind heavy metals to its cell surface performs as a protective mechanism for its cellular content (Aryal, 2021), potentially explicating its tolerance to high As levels. Wolfe-Simon et al. (2011) perceived vigorous growth of the *Halomonadaceae* bacterial strain GFAJ-1 in media supplemented with As. They advocated that this strain might use As as a replacement for phosphorus. Our study established a comparable trend with *S. arlettae*, signifying that this strain could potentially utilize As as a substitute to phosphorus.

In addition to its extraordinary As tolerance, *S. arlettae* also unveiled the power to augment the growth of sunflower plants and assist the accretion of higher amounts of As in their roots. Previous investigation has indicated that *S. arlettae* can generate metabolites allied with both growth promotion and stress response when open to high levels of chromate stress, stretching up to 900 ppm (Qadir et al., 2020). Sunflowers showed the capacity to establish a symbiotic association with the rhizobacterium *S. arlettae*, which led to an amplified accumulation of As in comparison to non-symbiotic sunflowers. The plant proficiently phytoextracted a substantial amount of the added As from the growth medium, with a bioconcentration factor (BCF) exceeding 1. Notably, despite of the initial introduction of arsenate was predominant, the accrued arsenic principally existed in the form of arsenite. This resulted in the host tissues holding advanced levels of the highly toxic As^+3^ species (Rosen and Liu, 2009) that can be efflux by the plant species to the xylem and then shoots for vacuolar sequestration (Zhao et al., 2009). Additionally, plants possess mechanisms for detoxifying As, such as the binding of As ions with phytochelatins and the involvement of siderophores in this process (Zhao et al., 2009). This indicates that the presence of rhizobacteria aided the sunflower seedlings in converting arsenate to arsenite and subsequently facilitating its removal from the cells (Yan et al., 2019).

It’s worth noting that sunflower seedlings associated with *S. arlettae* demonstrated robust net NAR and RGR when exposed to 100 ppm of arsenate. The unexpected nature of these results could potentially be attributed to two main factors: 1) the heavy metal may be biosorbed by *S. arlettae*, which has established colonization on the sunflower roots. This biosorption could act as a protective mechanism, safeguarding the plants from the detrimental effects of arsenate; 2) the bacteria might utilize As as an energy source to fuel its own growth, leading to a beneficial interaction where both the plant and the bacteria thrive in the presence of arsenate (Yan et al., 2019); 3) the sequestration of As within the vacuoles of host plants or by microbes through the action of phytochelatins and flavonoids (Jain et al., 2011; Asgari Lajayer et al., 2019). In this particular research, subjecting non-symbiotic sunflowers to stress resulted in diminished levels of reduced glutathione, a factor that contributes to the synthesis of phytochelatins (PCs) through the transpeptidation reaction catalyzed by phytochelatin synthase (Sriprang and Murooka, 2007; Yadav, 2010). The content of precursor compounds for phytochelatins (PCs) was consistently higher in seedlings associated with *S. arlettae*. As non-symbiotic seedling growth was inhibited in reply to the stress, rhizobacteria perform a distinct task in enhancing sunflower’s ability to withstand elevated levels of As. In this context, the combination of biotransformation and biosorption serves as an effective strategy utilized by the microorganism as a defense mechanism against the influx of As into the cells, thus evading its harmful impacts (Chandra and Banik, 2021; Qadir et al., 2022, 2023).

Symbiotic seedlings, however, employed a mechanism to accumulate approximately twice the amount of toxic As, yet they displayed no signs of As poisoning and maintained their normal growth. To elucidate the underlying mechanism in symbiotic sunflower, numerous essential metabolites that contribute to stress tolerance and growth enhancement were investigated. The synthesis of IAA was significantly inhibited in sunflower seedlings subjected to As stress, which is among the various factors limiting their growth. Reduced levels of IAA lead to less developed roots, thereby diminishing the host’s capacity to efficiently uptake essential nutrients (Meena et al., 2020; Luo et al., 2022). Deprivation of nutrients disrupts the metabolic profile of the host, rendering it more susceptible to unfavorable environmental conditions (Rivero et al., 2022). Conversely, under arsenate stress, symbiotic seedlings exhibited a significantly higher concentration of IAA, with a notable portion also being released into the root exudates. Previous observations of rice under As stress indicated that growth resumed after external administration of IAA, which concurrently hindered the translocation of As. The primary factor influencing As adsorption by the cell wall and diminishing its translocation in high IAA seedlings is the enhancement of hemicellulose synthesis, increased cell division, and improved root development (He et al., 2022). Lower levels of As improved lipid accumulation in non-symbiotic seedlings, whereas higher concentrations of the heavy metal had the opposite effect. However, this impact was counteracted in symbiotic sunflowers. In connection with this, non-symbiotic seedlings subjected to As exposure exhibited elevated levels of endogenous malonaldehyde. This indicates that As-induced lipid peroxidation resulted in membrane degradation, as evidenced by increased electrolyte leakage in As-stressed sunflowers (Panda et al., 2003).

Proline plays a critical function in enabling plants to counteract the impacts of heavy metal stress. It functions by chelating metals, enhancing the antioxidant defense system, facilitating signaling pathways, and serving as an osmolyte (Hayat et al., 2012). In the current investigation, sunflowers subjected to heavy metal-induced stress, particularly As, employed a strategy of producing and releasing higher levels of proline as a response to mitigate the detrimental impacts. This indicates that proline’s role alone was not entirely effective in shielding the seedlings from the adverse consequences of As exposure. This response was observed in both symbiotic and non-symbiotic sunflowers. Nevertheless, the elevated levels of proline in symbiotic sunflowers suggest its vital role when combined with various other previously identified factors, contributing to the plant’s effective resilience against stress.

Lignification showed an increase in the sunflower tissues without *S. arlettae* association after being subjected to metal stress. This heightened lignification could be linked to the initiation of the plant’s protective mechanism, serving as an initial protective barrier against the intrusion of harmful substances or pathogens. The intensified lignification process might lead to slower plant growth, consequently impacting both the NAR and RGR (Díaz et al., 2001; Jha, 2017). The interaction between the chosen strain and sunflower may have played a role in normalizing tissue lignification to ensure regular cell elongation and, consequently, normal plant growth.

The presence of brown patches on DAB-stained leaves of *S. arlettae*-free sunflower seedlings under As stress indicated the accumulation of ROS. However, even under 100 ppm of As stress, seedlings associated with *S. arlettae* exhibited normal levels of ROS generation. Symbiotic seedlings demonstrated higher DPPH free radical scavenging activity compared to non-symbiotic seedlings, potentially due to the robust antioxidant systems in the former. The DPPH scavenging activity can efficiently substitute for a plant’s natural antioxidant system (Rahman et al., 2015). It is imperative to stress that non-symbiotic sunflower seedlings exposed to As stress exhibited an increase in catalase, peroxidase, and APX activity. These enzymes play crucial roles in the enzymatic antioxidant system. In response to varying doses of As, non-enzymatic antioxidants like flavonoids and proline were also elevated in these seedlings. These findings underscore that symbiotic sunflower seedlings possess a more comprehensive antioxidative defense system compared to non-symbiotic ones. Moreover, the elevated levels of specific antioxidants, including catalase, APX, peroxidase, flavonoids, and proline, may not individually suffice to effectively manage As-induced stress. Successfully mitigating As stress, scavenging resulting ROS, and preventing oxidative stress necessitate the collective enhancement of all antioxidant components. This is due to the specific affinity of catalase, APX, and peroxidases for H_2_O_2_. It is worth noting that SOD is essential for the detoxification of superoxide radicals (Mittler, 2002). The study has revealed that maintaining optimal levels of H_2_O_2_ and superoxide radicals necessitates a delicate balance among the performances of assorted factors in the antioxidant system (catalase, SOD, and APX). Furthermore, apart from upholding this equilibrium, the sequestration of metal ions is believed to be crucial for minimizing the generation of the intensely deleterious hydroxyl radical (Mittler, 2002). The coordination among catalase, SOD, and APX is crucial for the regeneration of redox-active compounds like glutathione and ascorbate within the cellular redox system (Foyer and Noctor, 2000). The heightened glutathione content in symbiotic sunflowers within this chemical complex seems to have a significant impact in the process of As complexation and sequestration. This action occurs prior to the ultimate sequestration by phytochelatins (PCs), effectively preventing the heavy metal from reaching critical tissues (Foyer and Noctor, 2000).

One of the fundamental approaches for the remediation of heavy metal-contaminated soils involves enhancing their bioavailability to plants used for the purpose of efficient depletion from the soil (Li et al., 2018). This study documented the bio-transformation of metals by *S. arlettae*, rendering them bioavailable to the associated sunflower plants for active absorption from the soil. Furthermore, the accumulation of As primarily occurred in the roots, with limited translocation to the edible parts of the plants (Dinu et al., 2020). Plants treated solely with As actively accumulate the metals, resulting in inhibited growth, lower NAR, and RGR. In contrast, sunflower seedlings associated with *S. arlettae* exhibited increased uptake and accumulation of metals, coupled with improved NAR and RGR compared to control plants. This is indicative of the fact that *S. arlettae* enhances the host plants’ biomass, creating more space for As accumulation and translocation, while also preventing their toxic effects through detoxification and compartmentalization.

## 5 Conclusion

Considering the outcomes of this investigation, it can be inferred that the isolated *S. arlettae* performs a pivotal role as a PGPR in alleviating arsenate stress in *H. annuus*. This is achieved through the bio-transformation of As^+5^ to As^+3^. The presence of *S. arlettae* stimulates the advancement of host plant growth by fortifying the production of plant growth regulators and bolstering the antioxidant system. This enhancement involves the upregulation of antioxidant enzymes, which effectively counteract ROS and maintain cellular integrity. Given its abilities, the isolated *S. arlettae* could also serve as a promising candidate as biofertilization in As-polluted soils.

## Data availability statement

The original contributions presented in the study are included in the article/**Supplementary Material**. Further inquiries can be directed to the corresponding authors.

## Author contributions

MQ: Data curation, Formal analysis, Investigation, Methodology, Writing – original draft. AH: Conceptualization, Data curation, Project administration, Supervision, Validation, Writing – original draft, Writing – review & editing. MS: Conceptualization, Supervision, Validation, Writing – original draft. MH: Conceptualization, Project administration, Resources, Writing – review & editing. AI: Data curation, Writing – original draft, Writing – review & editing. MI: Methodology, Writing – review & editing. AA: Formal analysis, Methodology, Writing – review & editing. AFA: Funding acquisition, Resources, Writing – review & editing. SA: Formal analysis, Writing – original draft.
