# Supplementary material for: Staphylococcus arlettae mediated defense mechanisms and metabolite modulation against arsenic stress in Helianthus annuus
Source: Front Plant Sci. 2024 Jun 17;15:1391348. doi: 10.3389/fpls.2024.1391348 (PMC11216036; doi:10.3389/fpls.2024.1391348)
Supplement: Supplementary file 1 [file DataSheet_1.docx]

**
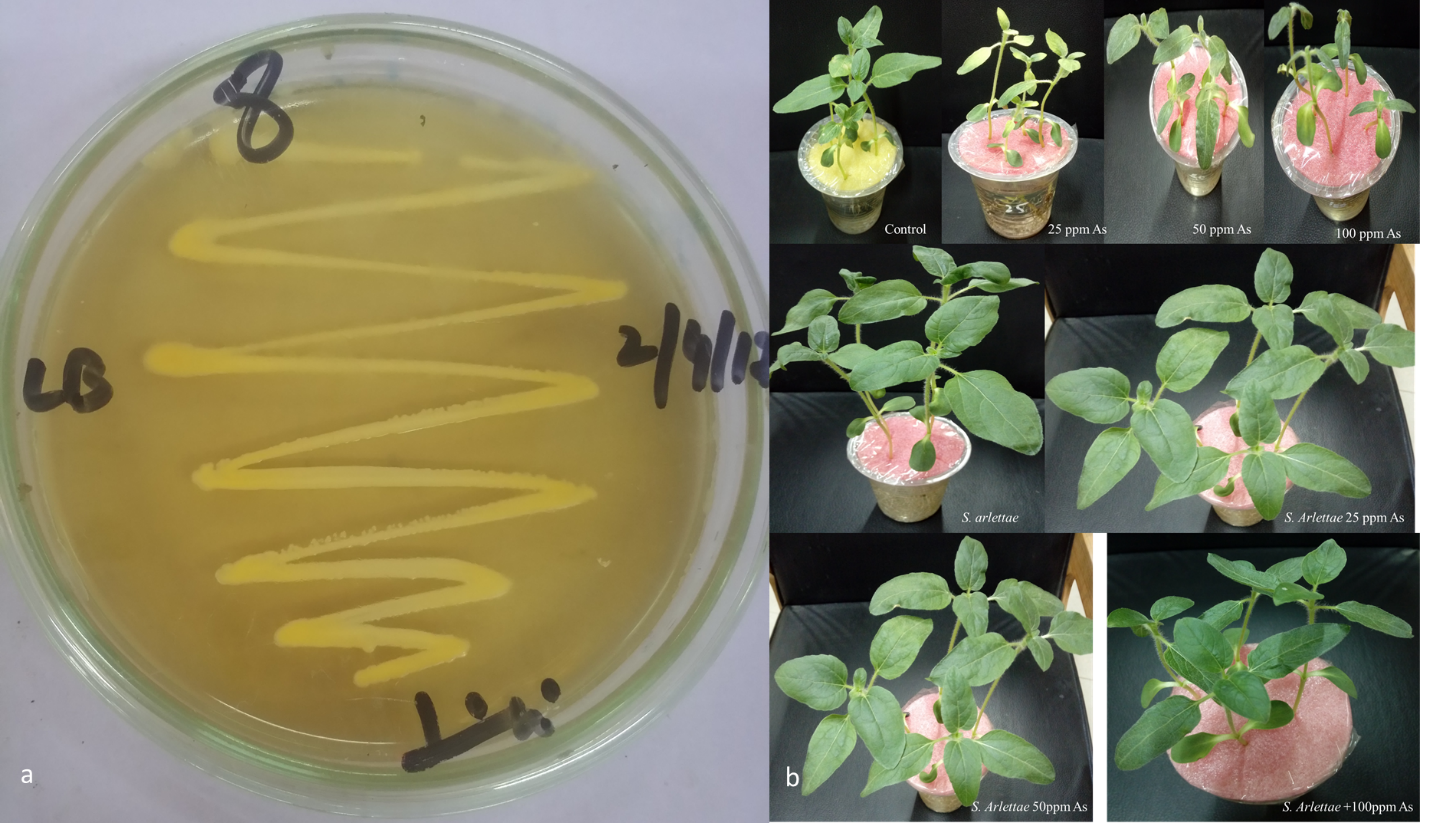
**

**Figure S1.** A pure colony of a) *Staphylococcus arlettae* b) *H. annuus* seedlings in arsenic stress and *S. arlettae* inoculation**s.** The plants were grown in the same condition for 20 days and harvested after the visual impairment after arsenate supplementation.
